# How communicating a diagnosis of polycystic ovarian syndrome (PCOS) impacts wellbeing: a retrospective community survey

**DOI:** 10.3399/BJGPO.2022.0014

**Published:** 2022-08-10

**Authors:** Jane Ogden, Lucy Bridge

**Affiliations:** 1 School of Psychology, University of Surrey, Guildford, Surrey, UK

**Keywords:** Polycystic ovary syndrome (PCOS), primary care, consultation, satisfaction, language, wellbeing, quality of life

## Abstract

**Background:**

Polycystic ovary syndrome (PCOS) is associated with wellbeing. Many women report dissatisfaction with the diagnostic process.

**Aim:**

This study assessed the impact of aspects of the diagnostic consultation on subsequent wellbeing.

**Design & setting:**

A retrospective community survey.

**Method:**

Females with PCOS (*n* = 147) completed measures of aspects of the diagnostic consultation (consultation satisfaction, language used in terms of framing and focus) and current wellbeing (body esteem, quality of life).

**Results:**

Most diagnoses took place in primary care. The majority showed a medium degree of satisfaction with the consultation. Most diagnoses were framed using a neutral term ‘raised’ but many used the more judgemental term ‘abnormal’. The majority focused on taking oral contraception and weight management. Poorer body esteem (body dissatisfaction and dieting behaviour) and poorer quality of life (self-identity, concerns about fertility, physical health, hirsutism, and overall quality of life) were predicted by lower communication comfort during the diagnostic consultation and greater use of the word ‘raised’. Greater use of the word ‘irregular’ predicted greater concerns about fertility, greater focus on fertility predicted greater concerns about physical health, and greater focus on appearance predicted greater concerns about hirsutism.

**Conclusion:**

How a diagnosis of PCOS is delivered can impact subsequent wellbeing. The diagnostic consultation may take a few minutes, yet how these minutes are managed, what words are used, and how this makes the patient feel may change how the patient makes sense of their condition and influence the impact of the condition on their wellbeing for the longer term.

## How this fits in

Women with PCOS rated their past diagnostic consultation and current wellbeing. Poorer communication comfort in the consultation predicted poorer current wellbeing. Poorer current wellbeing was predicted by greater use of the words ‘raised’ and ‘irregular’ and a focus on fertility and appearance. How a diagnosis of PCOS is given can have a longer-term impact.

## Introduction

PCOS is a common disorder affecting 12%–18% of women^1^ and resulting in a range of physical symptoms and metabolic issues.^2–6^ Women with PCOS are also more likely to experience depression, eating disorders, social anxiety, body dissatisfaction and diminished sexual satisfaction, and reduced quality of life.^7–13^ One possible explanation for this could be the patients’ experience of the diagnostic consultation in which they first have their condition explained by a healthcare professional (HCP), as this key moment has the capacity to shape how a person makes sense of their condition. Some research has addressed the impact of having the diagnosis of PCOS and has identified both benefits and harms.^14^ Other research has explored the experience of the diagnostic consultation using qualitative methods and indicates that women describe a range of issues including a lack of empathy, information, and advice from HCPs, difficulty in accessing specialists, unsatisfactory medication, insufficient help with fertility, and a lack of knowledge about comorbidities.^15,16^ Further, quantitative research indicates a lack of information giving in the consultation,^17^ feelings of distrust of the HCP,^18^ dissatisfaction with the diagnosis,^19^ and an inability to discuss mental health issues.^17^ Hoyos *et al* also found that 51.2% of participants were 'not satisfied’ with the recommended treatment options.^20^

Women with PCOS often experience poorer wellbeing. Many also report dissatisfaction with the diagnostic process. Research addressing a range of health conditions such as cancer, diabetes, pain, cardiovascular disease, and Parkinson’s disease indicates that patient satisfaction with the consultation can impact on health outcomes in the future.^21–25^ Research also indicates a role for the specific words used in the consultation and the way in which a diagnosis is made.^26–31^ To date, however, no research has specifically explored the impact of aspects of the diagnostic consultation for PCOS on women’s subsequent wellbeing. Therefore, the present study aimed first to describe the diagnostic consultation and second to assess the impact of aspects of the diagnostic consultation, with a focus on the effect of consultation satisfaction and language used on women’s subsequent wellbeing in terms of body esteem and quality of life.

## Method

### Participants

Women aged ≥18 years with a formal diagnosis of PCOS (*n* = 147) were recruited via social media, including support groups for women with PCOS.

### Design

A cross-sectional design to assess aspects of the diagnostic consultation and current wellbeing. Ethical approval was obtained from the University of Surrey Ethics Committee.

### Measures

Participants completed an online questionnaire assessing their demographics, HCP demographics, aspects of their diagnostic consultation, and current wellbeing. Reliability was assessed using Cronbach’s alpha where appropriate.

#### Participant demographics

Participants described their current age, age at diagnosis, delay to diagnosis, ethnicity, age of symptom onset, and family history of PCOS.

#### HCP demographics

The HCP at the diagnostic consultation was described in terms of gender, qualification (nurse, GP, consultant, gynaecologist), and if the setting was private or public health care.

#### Aspects of the diagnostic consultation

Aspects of the diagnostic consultation were assessed as satisfaction with the consultation and language during the consultation, with the latter assessed in terms of the framing of the problem and the focus of the solution.

##### Satisfaction with the consultation

Satisfaction with the consultation was assessed using the Medical Interview Satisfaction Scale (MISS-21),^32^ consisting of 3 subscales relating to distress relief (five items: for example '*the doctor has relieved my worries about my illnes*s'; *α* = 0.86), communication comfort (four items: for example '*I felt embarrassed while talking with the doctor*'; *α* = 0.72), and rapport (eight items: for example '*I really felt understood by my doctor*'; *α* = 0.93), rated on a 7-point Likert scale from 'very strongly disagree'^1^ to 'very strongly agree'.^7^

##### Language during the consultation

For framing of the problem, participants described the language used during their diagnosis for fertility problems (testosterone, periods, infertility); weight problems (weight); and appearance problems (acne, hirsutism, masculine characteristics), and recorded whether the HCP used the more neutral words ‘irregular’ and ‘raised’, and the more judgemental words ‘abnormal’ and ‘unusual’ for each problem, rated 'yes' or 'no'.

For the focus of the solution, participants were asked whether the HCP focused on appearance problems (acne, hair management/removal); fertility problems (recommend early conception, use of coil, use of implant, take oral contraceptives, use metformin); or weight problems (lose weight, manage weight, alter diet, cut out food groups [vegan, dairy free, gluten free, keto]), rated 'yes' or 'no'.

### Current wellbeing

Current wellbeing for the past 4 weeks was assessed as body esteem and body dissatisfaction; dieting behaviour; and quality of life.

#### Body esteem

This was assessed in terms of body dissatisfaction using the body shape questionnaire^33^ (10 items, for example '*has being with thin women made you feel self-conscious about your shape?*', *α* = 0.94). Items were rated from never^1^' to 'always'.^6^

#### Dieting behaviour

Participants rated their dieting behaviour using the restrained eating subscale of the Dutch eating behaviour questionnaire (DEBQ^34^; 10 items, for example '*do you try to eat less at mealtimes than you would like to ea*t', *α*=0.93). Items were rated from 'never^1^' to 'very often'.^5^

#### Quality of life

This was assessed using a brief version of the Health-Related Quality of Life questionnaire for PCOSQ, ^35^ ^36^ which consisted of 5 subscales: self identity (three items, for example *'lack of satisfaction with being a woman*', *α* = 0.80); fertility concerns (three items, for example '*felt concerned about infertility in the future*, *α* = 0.71); physical health concerns (three items, for example '*experienced concern about the long-term effects of PCOS medication*', *α* = 0.61); sexual function concerns (three items, for example '*experienced loss of libido because of PCOS*', *α* = 0.87); and hirsutism concerns (three items, for example '*embarrassed about having excess body hair*', *α* = 0.95). Items were rated from ‘not at all’^1^ to 'very much'. A total quality of life score was also computed.

### Procedure

Participants were recruited via social media and provided a link to complete the information sheet, consent form, and the 15 minute online survey.

### Data reduction and analysis

For descriptive purposes, the three subscales of consultation satisfaction were recoded into low (1–3.5), medium (3.6–4.5), and high (4.6–7). Mean scores were also computed for the subscales of consultation satisfaction and current wellbeing, with higher scores reflecting greater distress relief, greater communication comfort and greater rapport, greater body dissatisfaction and dieting behaviour, and poorer quality of life in terms of poorer self-identity, and greater concerns about fertility, physical health, sexual function, and hirsutism, and poorer overall quality of life. For language used in the consultation, the framing of the problem was analysed by summating the scores for the four words ‘abnormal’, ‘unusual’, irregular’, and ‘raised’. For focus on the solution scores were summated for each problem area ‘appearance’, ‘fertility’, and ‘weight’. Higher scores reflected greater frequency of these words or areas. Data were analysed to describe participant and HCP demographics, consultation satisfaction, and the language used in the consultation using descriptive statistics. The role of participant and HCP demographics, consultation satisfaction, and language used in predicting patient current wellbeing (body esteem and quality of life) was assessed using multiple regression analyses. All analyses used Jamovi (version 2.2.5).

## Results

### Participant and HCP demographics

Mean age was 27 years (range 18–62 years) and the majority described themselves as White. The mean age of diagnosis was 21 years, the mean delay until diagnosis was 3.38 years (range 0–12 years), and the most reported symptom onset was between 10 and 15 years old. The majority had no family history of PCOS. The majority of participants had been diagnosed by a female HCP, although just under half had seen a male HCP. The majority had been diagnosed by a GP, although a large minority had been diagnosed by a gynaecologist. The majority of diagnoses had taken place in a public rather than private sector setting (see Table 1 ).

**Table 1.** Participant and HCP demographics

Participants’ demographics	
Current age	Mean = 27 (SD = 8.1)Range 18–71
Family history of PCOS	Yes = 38 (25.9%)No = 109 (74.1%)
Age at diagnosis	Mean = 21.2 (SD = 5.3)Range = 14–38
Years since diagnosis	Mean = 5.8 (SD = 7.3)Range = 0–42
Delay to diagnosis	Mean = 3.38 (3.1)Range = 0–12
Ethnicity	White = 124 (84.4%)
	Black = 2 (1.4%)
	Asian = 10 (6.8%)
	Other = 11 (7.5%)
Age of symptom onset	10–15 = 71 (48.2%)
	16–20 = 51 (34.7%)
	21–25 = 16 (10.9%)
	26–30 = 4 (2.7%)
	≥31 = 5 (3.4%)
**HCP demographics at diagnostic consultation**	
Gender^a^	Male = 60 (41.4%)
	Female = 85 (58.6%)
Healthcare setting^a^	Private = 33 (22.8%)
	Public = 112 (77.2%)
Healthcare profession^a^	Nurse = 5 (3.4%)
	GP = 68 (46.9%)
	Gynaecologist = 50 (34.5%)
	Consultant = 22 (15.2%)

HCP = healthcare professional. SD = standard deviation.

^a^Missing data *n* = 2.

### Describing the consultation

The consultation was described in terms of consultation satisfaction and the language used.

#### Consultation satisfaction

A majority of participants (>50.0%) showed a medium degree of satisfaction with their diagnostic consultation in terms of the three subscales: distress relief, communication comfort, and rapport. A large minority (>25%) were not satisfied with distress relief or rapport (Table 2).

**Table 2.** Describing the consultation—consultation satisfaction

	Low(1–3.5)	Medium(3.6–4.5)	High(4.6–7.0)	Mean (SD)
Distress relief	53 (36.8%)	74 (51.4%)	17 (11.8%)	3.3 (1.3)
Communication comfort	21 (14.6%)	85 (59.0%)	38 (26.4%)	4.11 (1.2)
Rapport	38 (26.4%)	75 (52.1%)	31 (21.3%)	3.89 (1.4)

Missing data *n* = 3. SD = standard deviation.

#### Language used in the diagnostic consultation

Language used was assessed in terms of framing of the problem and focus of the solution.

##### Framing of the problem

The more neutral term ‘raised’ was used the most frequently to describe all components of weight and appearance related problems and most components of fertility. The term 'irregular', however, was used the most frequently to describe periods. The more judgemental term ‘abnormal’ was used the next most frequently for all aspects of fertility, weight, and appearance problems, apart from when describing ‘infertility’ (see Table 3).

**Table 3.** Describing the consultation language used during diagnosis: framing of the problem

	Abnormal, *n*	Unusual, *n*	Irregular, *n*	Raised, *n*
**Fertility**				
Testosterone	36	7	16	**68** ^a^
Periods	26	6	**117** ^a^	10
Infertility	15	13	19	**33** ^a^
**Weight**	19	6	14	**69** ^a^
**Appearance**				
Acne	24	6	11	**48** ^a^
Hirsutism	25	12	1	**33** ^a^
Masculine characteristics	12	11	2	**39** ^a^
Total frequency	157	61	180	**300** ^a^

^a^Denotes most frequent use.

##### Focus of the solution

The majority ( >50%) of consultations had focused on taking oral contraception, losing weight, managing weight, and altering diet. Only a minority (<30%) had focused on acne medication, hair management/removal, or using a coil or implant for contraception. The most common focus was on taking oral contraception, followed by 3 weight management strategies (lose weight, manage weight, alter diet), as shown in Table 4.

**Table 4.** Describing the consultation language used during diagnosis: focus of the solution

Focus of solution		Yes, *n/N (*%)	Rank**^a^**
**Appearance**	Acne medication	37/142 (26.1)	8
	Hair management/removal	35/144 (24.3)	9
**Fertility**	Metformin	46/144 (31.9)	6
	Use of implant	18/142 (12.7)	11
	Use of coil	29/142 (20.3)	10
	Early conception	50/143 (35)	5
	Oral contraception	102/145 (70.3)	1
**Weight management**	Lose weight	90/143 (62.9)	2
	Manage weight	86/144 (59.7)	4
	Alter diet	87/144 (60.4)	3
	Cut out food groups	43/143 (30.1)	7

^a^1 = most frequent, 11 = least frequent.

### The role of aspects of the diagnostic consultation in predicting body esteem and quality of life

The results were then analysed to explore the role of participant and HCP demographics and aspects of diagnostic consultation in predicting current wellbeing using multiple regression analysis. For this analysis, HCP qualification was dichotomised into non-specialist (nurse and GP) and specialist (gynaecologist and consultant) (see Table 5).

**Table 5.** Role of aspects of the consultation in predicting current wellbeing

	Body esteem		Quality of life					
	Body diss(β, p)	Rest eating(β, p)	Self identity(β, p)	Fertility concern(β, p)	Sexual function(β, p)	Physical health(β, p)	Hirsutism(β, p)	Total quality of life(β, p)
**Demographics**								
Current age	–0.150.14	–0.140.16	–0.180.07	–0.080.44	0.0090.92	–0.140.13	0.080.37	–0.060.49
Diagnosis age	0.140.15	0.030.78	**0.23** **0.01** ^a^	0.170.07	0.150.11	0.130.13	–0.070.41	0.160.06
HCP gender	0.170.06	0.020.85	0.140.11	0.100.23	0.030.75	0.030.71	0.120.13	0.110.16
HCP qual	–0.060.5	–0.060.53	–0.150.08	–0.070.44	–0.0010.99	–0.130.11	–0.100.2	–0.130.11
Model 1 adj R^2 /^F/ p	0.005/1.17/0.3	0.003/1.12/0.35	0.03/2.16/0.08	–0.002/0.94/0.44	–0.008/0.72/0.6	0.02/1.89/0.12	–0.01/0.31/0.8	–0.003/0.88/0.48
**Communication satisfaction**								
Distress relief	–0.190.16	–0.070.59	–0.090.43	–0.090.45	–0.120.35	–0.010.92	–0.140.22	–0.150.19
Comm comfort	–0.260.03^a^	**–0.26** **0.03** ^a^	**–0.24** **0.02** ^a^	**–0.27** **0.01** ^a^	–0.120.29	**–0.33** **0.001** ^a^	**–0.29** **0.004** ^a^	**–0.33** **0.001** ^a^
Rapport	0.210.15	0.150.15	0.110.43	0.140.31	0.090.31	0.170.19	**0.26** **0.04** ^a^	0.230.07
Model 2 adj R^2^/ F/p	**0.07/2.52/0.02** ^a^	**0.05/2.01/0.06** ^a^	**0.09/3.23/0.003** ^a^	**0.07/2.48/0.02** ^a^	0.003/1.37/0.18	**0.11/3.54/0.002** ^a^	**0.07/2.48/0.02** ^a^	**0.12/3.86/0.001** ^a^
**Framing and focus of language used**								
Irregular	–0.0050.9	–0.020.84	–0.010.87	**0.21** **0.01^a^**	0.090.31	0.130.09	–0.070.38	0.070.35
Raised	**0.28** **0.007** ^a^	0.150.15	**0.25** **0.009** ^a^	**0.16** **0.08** ^a^	**0.26** **0.008** ^a^	**0.26** **0.004** ^a^	**0.33** **0.001** ^a^	**0.37** **0.001** ^a^
Abnormal	0.060.52	0.050.61	0.090.24	–0.020.79	–0.0040.96	0.110.18	0.120.12	0.110.18
Unusual	–0.0060.94	–0.110.22	–0.120.14	0.00010.99	–0.010.87	–0.050.51	–0.110.14	–0.090.20
Appearance	–0.080.38	–0.050.59	0.070.38	–0.040.67	0.010.87	–0.020.77	**0.17** **0.03** ^a^	0.080.33
Fertility	0.060.5	0.090.34	0.040.65	0.130.13	0.0080.93	**0.18** **0.02** ^a^	0.040.61	0.090.23
Weight	–0.0030.9	0.120.21	–0.040.65	0.030.77	–**0.21** **0.03** ^a^	0.010.90	0.040.68	–0.070.39
Final Model adj R^2^/F/ p	**0.10/2.06/0.02** ^a^	**0.07/1.77/0.05** ^a^	**0.15/2.76/0.001** ^a^	**0.12/2.40/0.005** ^a^	0.03/1.37/0.17	**0.22/3.92/0.001** ^a^	**0.24/4.3/0.001** ^a^	**0.27/4.73/0.001** ^a^

^a^ *P*<0.05

Body diss = body dissatisfaction. Comm comfort = communication comfort. HCP = healthcare professional. Rest eating = restrained eating.

#### Predicting body esteem

Greater current body dissatisfaction was predicted by lower communication comfort in the diagnostic consultation, with more frequent use of the word ‘raised’ accounting for 10% of the variance. Greater restrained eating was predicted by lower communication comfort with the diagnostic consultation, accounting for 7% of the variance.

#### Predicting current quality of life

Poorer current self-identity was predicted by being older at diagnosis, lower communication comfort with the diagnostic consultation, lower depression, and greater use of the word ‘raised’, accounting for 15% of the variance. Greater current concern about fertility was predicted by lower communication comfort during the diagnostic consultation and more frequent use of the words ‘irregular’ and ‘raised’, accounting for 12% of the variance. None of the models were significant for predicting sexual function. Increased physical health concern was predicted by reduced communication comfort with the consultation, increased use of the word ‘raised’, and increased focus on fertility, accounting for 22% of the variance. Increased concerns for hirsutism were predicted by lower communication comfort but greater rapport, more frequent use of the word ‘raised’, and greater focus on appearance, accounting for 24% of the variance. Total poorer quality of life was predicted by lower communication comfort and more frequent use of the word ‘raised’ in the diagnostic consultation, accounting for 27% of the variance.

### Discussion

### Summary

This study aimed to describe the diagnostic consultation for PCOS and to assess the impact of aspects of this consultation on wellbeing. Descriptive analysis indicated that the majority of diagnoses had been delivered by a female HCP in primary care. The majority of participants reported a medium degree of satisfaction with the consultation in terms of distress relief, communication comfort, and rapport, although a large minority reported dissatisfaction with distress relief and rapport. The more neutral word ‘raised’ was most commonly used to frame problems relating to fertility, weight, and aspects of appearance; the word ‘irregular’ was most commonly used to describe periods; and the more judgemental word ‘abnormal’ was the second most commonly used word for most problems. Most consultations focused on solutions relating to oral contraception and weight management. In terms of predicting outcomes, communication comfort consistently predicted wellbeing in terms of body dissatisfaction, restrained eating, self-identity, concerns about fertility, physical health, and overall quality of life, with poorer communication comfort in the diagnostic consultation predicting lower wellbeing for all these components. In terms of specific words used, greater frequency of the word ‘raised’ was related to poorer wellbeing in terms of all components apart from restrained eating, and greater frequency of the word ‘irregular’ was related to greater concerns about fertility. Further, a focus on appearance related to greater concerns about hirsutism and a focus on fertility related to greater concerns about physical health.

### Strengths and limitations

There are some methodological limitations that need to be considered. Primarily, the data concerning the consultation were retrospective and therefore open to issues of bias, limiting conclusions about causality. Future research should use a longitudinal design to follow patients up after their consultation or, ideally, use an experimental design that allows for manipulation of the content of a consultation and evaluation of the longer-term impact on patient health outcomes. Second, participants were recruited via social media, which may alter the kinds of patients that take part. Future research could recruit via a hospital or primary care setting to access a more representative sample of patient receiving their diagnosis of PCOS. The present study, however, was the first to show that what occurs within this brief interaction has a long-lasting effect on the patient.

### Comparison with existing literature

Previous research suggests that PCOS can impact on women’s wellbeing^8–11^^,^^13^ and that women can be dissatisfied with the diagnostic process.^15, 20^ The results from the present study indicate that these two factors are related, reflecting existing research that demonstrates consultation satisfaction plays a key role on health outcomes in other conditions.^21–25^ At a more specific level, the results also highlight the role of language, in concordance with research across a number of health contexts that indicates that individual words can change how patients make sense of their condition.^26–31^ Specifically, the more neutral words ‘raised’ and ‘irregular’ were more predictive of poorer wellbeing than the more judgemental words ‘abnormal’ and ‘unusual’.

### Implications for practice

A diagnosis of PCOS can have a significant impact on the lives of patients. But how this diagnosis is delivered is also key. Practitioners should therefore ensure that they take time to put their patient at ease during these diagnostic consultations and make them feel comfortable with what is being communicated. They should also be aware that both the individual words they use and what they chose to emphasise as ways to manage PCOS could have a negative and longer lasting impact on their patients. While the diagnostic consultation may only take a few minutes, the results from the present study indicate that how these minutes are managed, what words are used, what is focused on, and how this makes the patient feel may change how the patients makes sense of their condition and influence the impact of the condition on their wellbeing for the longer term.
